# Mechanism of inulin in colic and gut microbiota of captive Asian elephant

**DOI:** 10.1186/s40168-023-01581-3

**Published:** 2023-07-06

**Authors:** Tingbei Bo, He Liu, Min Liu, Qiyong Liu, Qingduo Li, Yipeng Cong, Yi Luo, Yuqi Wang, Bo Yu, Tianchun Pu, Lu Wang, Zheng Wang, Dehua Wang

**Affiliations:** 1 School of Grassland Science, Beijing Forestry University, Beijing, 100091 China; 2 State Key Laboratory of Integrated Management of Pest Insects and Rodents, Institute of Zoology, Chinese Academy of Sciences, Beijing, 100101 China; 3 Beijing key laboratory of captive wildlife technology, Beijing Zoo, Beijing, 100044 China; 4 CAS Center for Excellence in Biotic Interactions, University of Chinese Academy of Sciences, Beijing, 100049 China; 5 State Key Laboratory of Infectious Disease Prevention and Control, National Institute for Communicable Disease Control and Prevention, Chinese Center for Disease Control and Prevention, Beijing, China; 6 Department of Vector Control, School of Public Health, Shandong University, Jinan, 250012 China; 7 School of Life Science, Shandong University, Qingdao, 266237 China

**Keywords:** Inulin, Colic, Gut microbiota, Asian elephants (*Elephas maximus* Linnaeus)

## Abstract

**Background:**

Gut microbiota have a complex role on the survivability, digestive physiology, production, and growth performance in animals. Recent studies have emphasized the effects of prebiotics therapy on the gut disease, but the relationship between elephant gut-related diseases and prebiotics remains elusive. Here, a case study was undertaken to evaluate the mechanism of inulin treatment in colic in Asian elephant (*Elephas maximus* Linnaeus).

**Methods:**

Fecal samples were collected from a sick elephant and four healthy elephants. Analysis of microbial profile was carried out by 16S rRNA sequencing, and the short chain fatty acids were tested by gas chromatography. The physiological function of “inulin-microbiota” of elephant was verified in mice by fecal microbial transplantation (FMT). The expression of related proteins was determined by Western blotting and qPCR.

**Results:**

(1) Eating inulin can cure gut colic of the sick elephant and changed gut microbiota. (2) It was found that “inulin microbiota” from the post-treatment elephants can promote the proliferation of intestinal cells, increase the utilization of short chain fatty acids (SCFAs), maintain intestinal barrier, and reduce the inflammation in mice. (3) The mechanism was inulin—gut microbiota—SCFAs—immune barrier.

**Conclusions:**

Inulin contributed to rehabilitate the gut microbiota and gut immune barrier of the elephant with colic. This provides reasonable verification for using prebiotics to treat the colic in captive elephants.

Prebiotics will foresure play an increasingly important role in disease prevention and treatment of captive animals in the future.

**Graphical Abstract:**

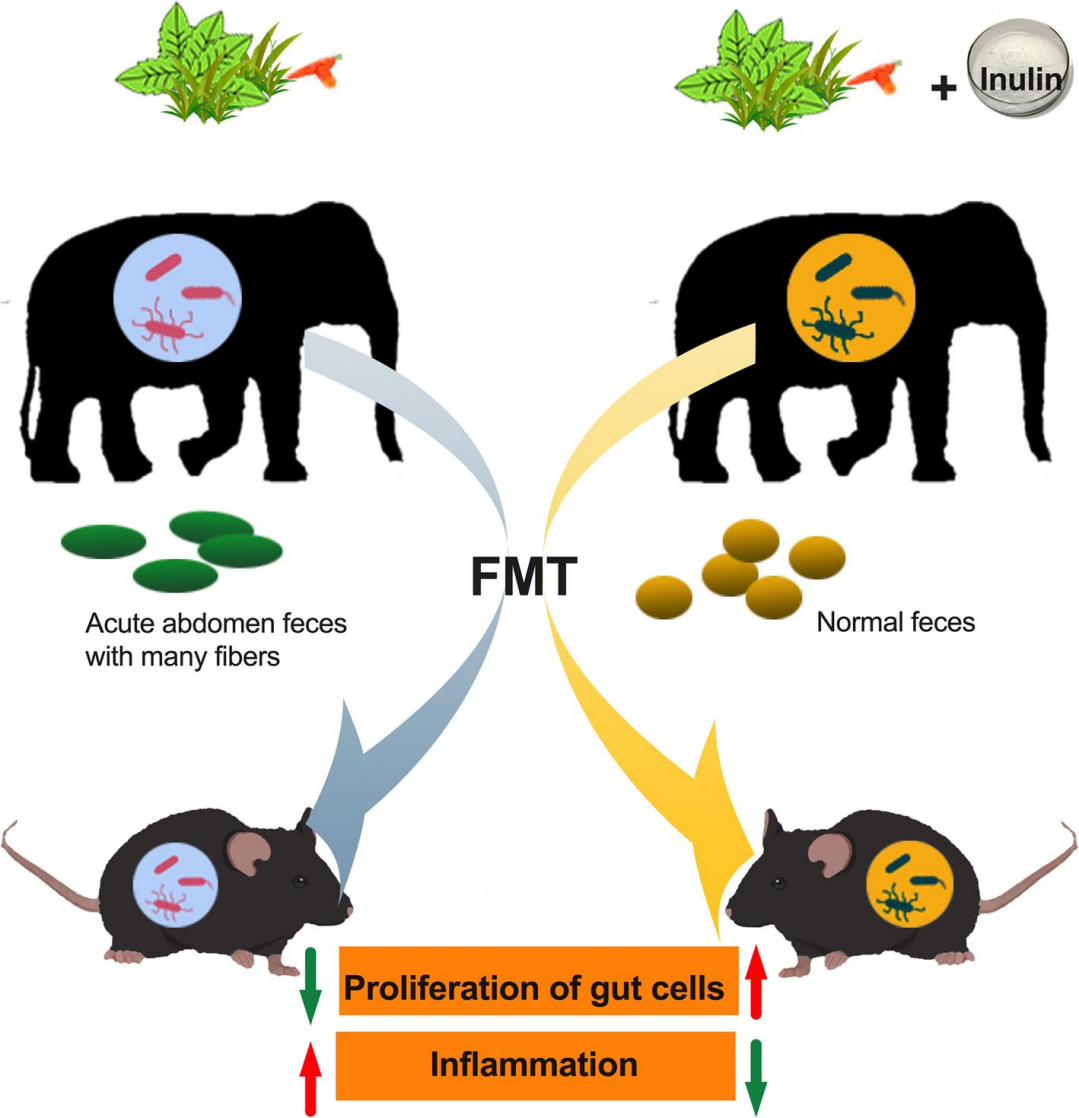

Video Abstract

**Supplementary Information:**

The online version contains supplementary material available at 10.1186/s40168-023-01581-3.

## Background

Gut microbiota have complex effects on the survival and reproduction of wild animals [1], and they are e ssential for host adaptation to their habitat and are associated with health and disease status [2, 3]. Environmental factors also affect the composition and structure of the gut microbiota of wild animals [4]. A recent study showed that alpha and beta diversity of the gut bacteria change in captive hosts [5, 6]. There are many reasons for the changes in gut microbiota under captive conditions, like space constraints, artificial diets, loss of companions, repressed emotions, and antibiotic resistance. Limited exposure to natural microbial sources was the key factor for changing gut microbiota [7] in Red Colobus Monkey (*Procolobus gordonorum*) and Andean bear (*Tremarctos ornatus*) [8, 9]. Therefore, changes in gut microbiota would lead to many diseases of captive animals.

Antibiotic resistance is a common problem in the treatment of captive animals. Studies have shown that increased interaction with humans leads to horizontal gene transfer of antibiotics and other bacteria increased in the living environment of captive animals (such as air and water) [10-12]. Therefore, reducing antibiotic use or providing prebiotic supplements to captive animals can alleviate changes in the microbiota [13]. The use of prebiotics may have a significant impact on the absorption and utilization of feed and health of various animals. Prebiotics are called indigestible carbohydrates, which regulate gut microbiota and gut environment, accelerate cellulose decomposition, and promote digestion of ruminants [14]; improve the body bone density; and improve the bone [15]. Prebiotics such as fructooligosaccharide and maltooligosaccharide have been commercialized and applied to the breeding of animals such as cattle, pigs, and chickens [16, 17]. In terms of disease, prebiotics’s efficacy in treatingdiarrhea has been confirmed in captive economic animals [17]. Inulin is a fructo-oligosaccharide type prebiotic, mainly derived from plants, but inulin does not usually exist in humans and animals, so it cannot be decomposed [18]. Inulin promotes the proliferation of beneficial bacteria (like *Bifidobacterium* and *Lactobacilli*), reduces harmful bacteria, and protects host health [18]. Studies have shown that the diet supplemented with inulin can more effectively alleviate colic in mice [19] and inhibits intestinal barrier dysfunction caused by obesity [20]. However, the effect of inulin on intestinal diseases in captive big mammals was unclear.

The Asian elephant (*Elephas maximus*) is listed as endangered (EN) by the IUCN (https://www.iucnredlist.org). They use complex intestinal microbial communities to ferment cellulose [21, 22]. Asian elephants often suffer from colic, constipation, intestinal blockage, or other gastrointestinal diseases after being kept in captivity [23]. In this study, we want to explore whether prebiotics (inulin) can cure abdominal colic in elephant and improve its gut microbiota. In addition, the mechanism of inulin in treating colic should be explored.

## Material and methods

### Elephants

Five adult Asian elephants in Beijing Zoo, named YINAN, ALALIYA, ZANLAN, WANGNANJIAO, and MIGAILA. Their food composition and nutrients are shown in Supplementary Tables S1 and S2. YINAN was suffered by persistent colic. The clinical symptoms are as follows: less alertness; leaned wall and reduced movements of the trunk, ears, tail, and legs; rising and lying frequently; and nose weakness. Reduced feed intake and fecal ball were larger and loose, which contains a large amount of undigested plant fiber, and less fecal quantity. Feces are shapeless, with a small amount of mucus on the surface, dark in color. Feces contained a large number of undigested grass materials, and the length of undigested grass was 10–20 cm. The urine and feces were collected to check parasitic and bacterial infections, and there were no parasites and the occult blood or sanies (Supplementary Reports).

### Fecal sample collection

On October 16, 23, and 24, the feces of four normal elephants were collected, and all the above 12 samples were taken as the control group (Con). We collected feces of YINAN for 3 days at October 15–17 as the pre-inulin group (3 samples), and then, yinan received dietary inulin (500 g per day, for 7 days) from October 18. After the recovery, the collected feces of YINAN for three times at October 22, 23, and 24 as the post-inulin group (3 samples). See Supplementary Table S3 for specific sampling dates. All stool samples were immediately frozen and stored at − 80 °C.

### Animals

**Fig. 1:**
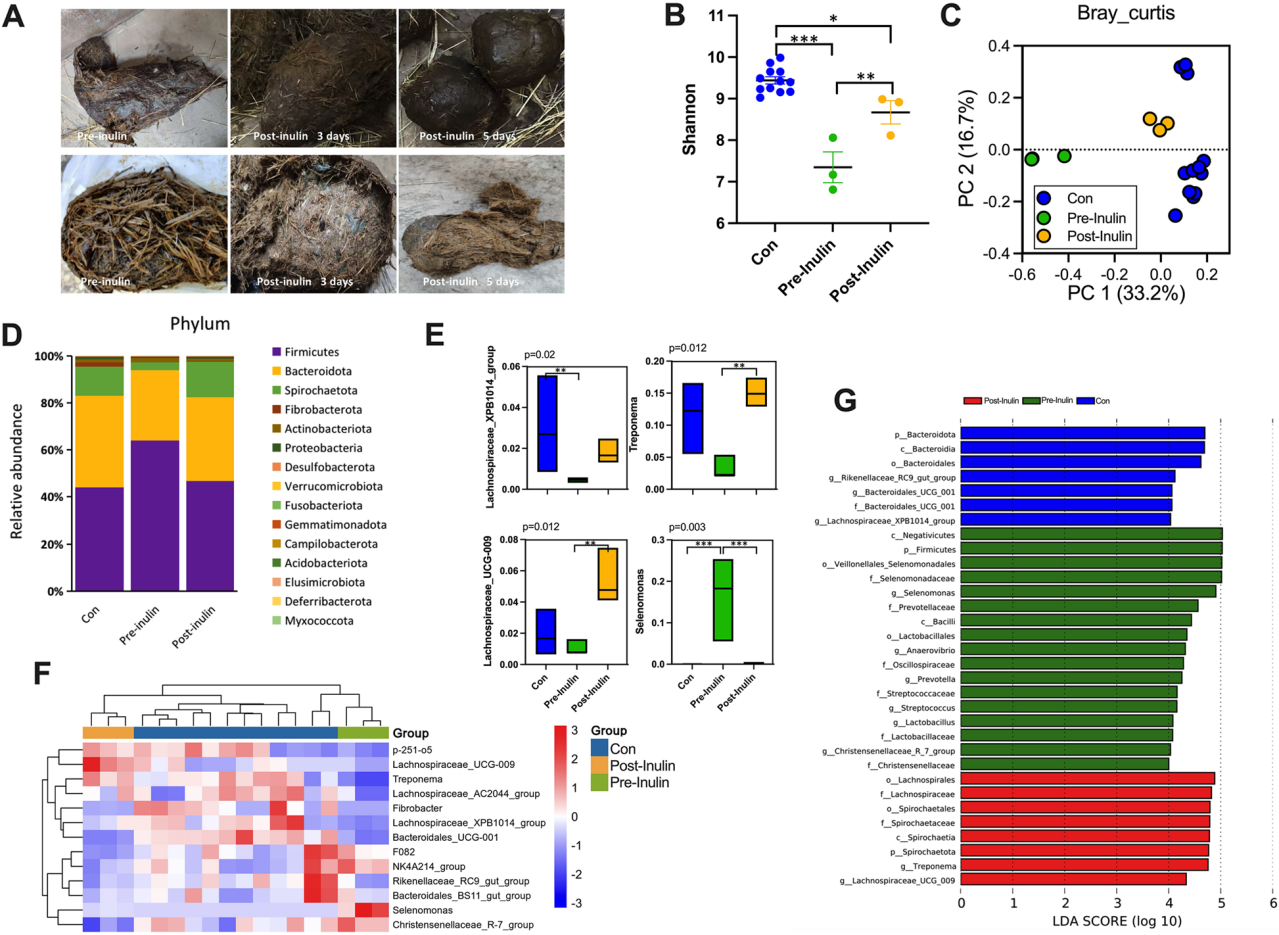
Inulin altered the gut microbiota of the sick Asian elephant. **A** Elephant fecal photos before and after treatment. **B** Alpha diversity (Shannon) of bacterial communities across groups. **C** PCoA plot based on Bray–Curtis distance metrics in different groups (ANOSIM). **D** Abundance represented as the proportions of ASVs classified at the phylum rank. **E** Relative abundance of specific genus significantly altered by inulin. **F** Cluster heatmap showing the proportions of ASVs classified at the genus rank. **G** Differential bacterial taxonomy selected by LEfSe analysis with LDA score > 4. Con: control group of 4 healthy elephants; Pre-inulin: before inulin treatment of the sick elephant; Post-inulin: after inulin treatment of the sick elephant. Data are means ± SEM

In experiment 2, C57BL/6 J mice were used as experimental animals. C57BL/6 J mice (21 days old) were bought from the SPF Biotechnology Co., Ltd. (Beijing, China). The mice were housed individually in a plastic cage and were maintained at room temperature (23 °C), under a photoperiod of 16L:8D. After the transplantation experiment, the mice were killed by carbon dioxide and the materials were taken. All animals were licensed under the Animal Care and Use Committee of the Institute of Zoology at the Chinese Academy of Sciences (Fig. 1).

### Fecal microbiota transplant (FMT)

**Fig. 2:**
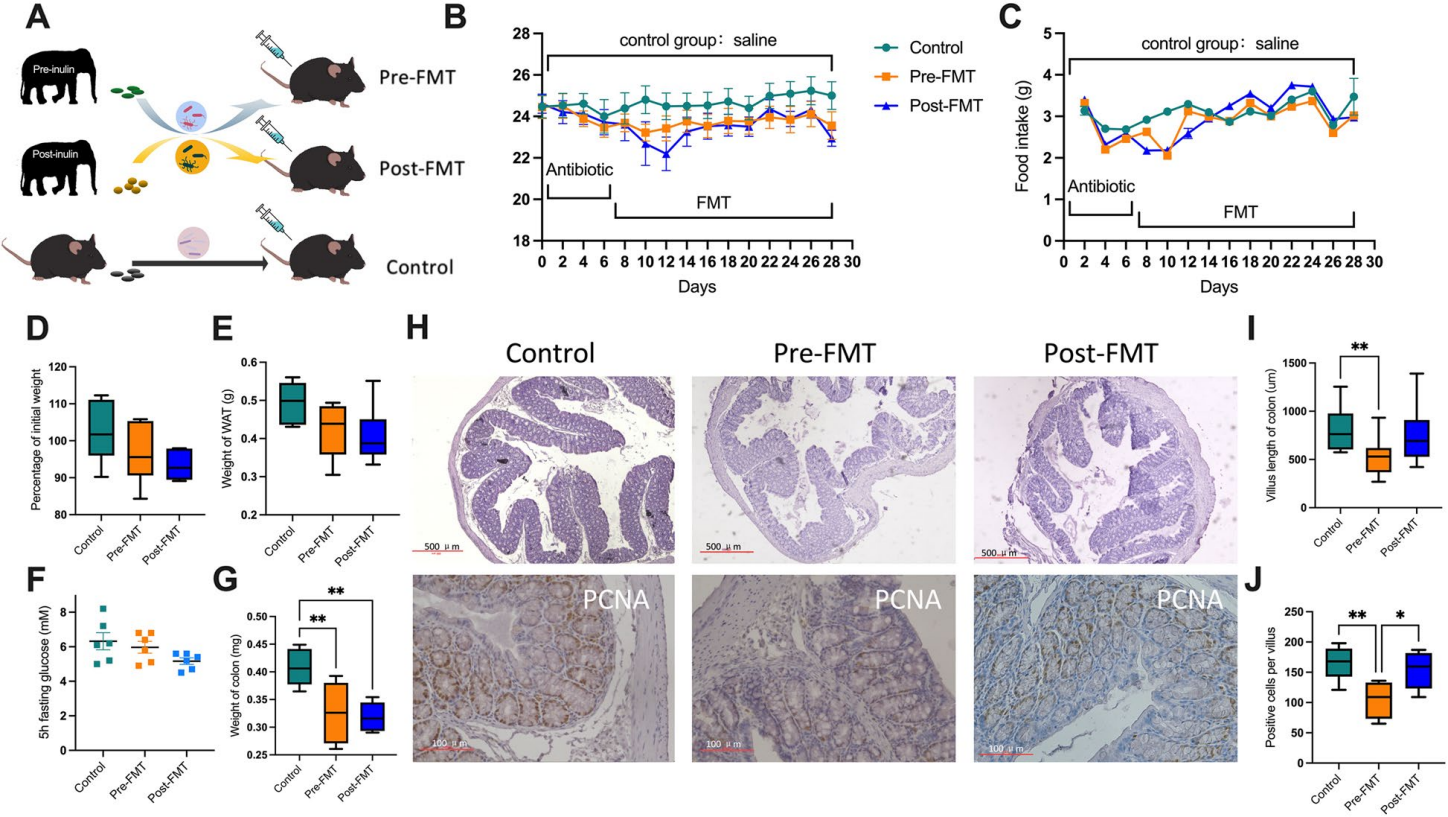
“Inulin-microbiota” affects energy metabolism in mice by FMT. **A** Pattern of fecal transplantation. **B** Changes of the body weight with time of transplantation (repeated measures ANOVA). **C** Changes of food intake with time of transplantation (repeated measures ANOVA). **D** Percentage of initial body weights. **E** Weight of WAT. **F** Five hours fasting glucose. **G** Weight of the colon. **H** Colon histopathologic appearance by H&E staining. Scale bars, 500 μm. Visualization of PCNA-positive cells in proximal colon by staining. Scale bars, 100 μm. **I** Colon villus length. **J** PCNA-positive cells per villus. Control: mice gavaged with mice fecal microbiota, Pre-FMT: mice gavaged with fecal microbiota from elephant before inulin treatment; Post-FMT: mice gavaged with fecal microbiota from elephant after inulin treatment. Data are means ± SEM. *P < 0.05, **P < 0.01, and ***P < 0.001

The experiment 2 used the fecal microbiota transplantation to identify the role of inulin (Fig. 2A). To remove microbiota, the healthy C57BL/6 J mice were housed at a room temperature (23 ± 1 °C) and were intragastric gavaged with fresh composite antibiotics (200 µL/day, containing 100 µg/mL neomycin, 50 µg/mL streptomycin, 100 U/mL penicillin; Sigma, Germany) for 6 days [24]. For microbiota transplantation, the feces were collected from YINAN before and after inulin treatment and diluted (200 mg) in 0.9% physiological saline (2 mL), and then, a 200-µL suspension was delivered by intragastric gavage to each bacteria-restricted recipient mice (pre-FMT and post-FMT). For the control group, mice feces suspension (200 µL) was delivered by intragastric gavage to each mouse (*n* = 6).

### 16S rRNA gene sequencing analysis

Bacterial DNA was isolated from the elephant and mice fecal samples using a DNeasy PowerSoil kit (Qiagen, Hilden, Germany). PCR amplification of the V3-V4 hypervariable regions of the bacterial 16S rRNA gene was carried out using universal primer pairs (343F: 5′-TACGGRAGGCAGCAG-3′; 798R: 5′-AGGGTATCTAATCCT-3′). Sequencing was performed on an Illumina Miseq with two paired-end read cycles of 300 bases each (Illumina Inc., San Diego, CA; OE biotech Co., Ltd., Shanghai, China).

Using Cutadapt software, cut out the primer sequence of the raw data sequence [25]. Using DADA2, the qualified double ended raw data in the previous step was used to perform quality filtering, noise reduction, splicing, and de chimerism quality control analysis using QIIME2 (2020.11) default parameters, and representative sequences and ASV abundance tables are obtained [26]. The database address was as follows: Silva (bacteria, eukaryotes)– https://ftp.arb-silva.de. At each classification level, the number of ASV and annotation results of each sample were counted. The microbial diversity was estimated using the alpha diversity that includes chao1 and Shannon. The Bray–Curtis distance matrix performed by QIIME2 was used for principal coordinates analysis (PCoA). Linear discriminant analysis (LDA) combined with effect size measurements was used to analyze and reveal the composition of different species in the three groups of biological communities.

### Hematoxylin and eosin staining

Mouse colons were fixed in 4% buffered paraformaldehyde at room temperature before embedding in paraffin. Tissues were sectioned at 5–6-µm thickness and stained with hematoxylin and eosin (H&E) staining. The colonic villus length in the colon was determined using ImageJ software from H&E [27].

### Immunohistochemistry

The tissue sections after antigen repaired were washed in PBS and blocked with goat serum, and the proximal colon section was incubated with rabbit anti-PCNA (D3H8P, Cell Signaling). Immunoreactivity was identified using biotinylated goat anti-rabbit immune globulin G (1:3000; Jackson ImmunoResearch). Sections were then incubated with ABC reagents (PK-6100; Vector Labs) for 60 min. Immunoreactive sites were subsequently identified by 3,3 diaminobenzidine (DAB substrate kit; Vector Labs), and tissue sections were photographed using a Nikon optical microscope. The number of PCNA positive cells per crypt in the proximal colon was counted.

### Measurement of protein expression by Western blot

The total protein from hypothalamus and colon were separated by SDS-PAGE and then transferred to PVDF membrane. The membrane was soaked with skimmed milk powder to reduce the binding of non-specific antibodies. The membranes were then exposed to primary antibodies and secondary antibodies (peroxidase-conjugated goat anti-rabbit IgG (111–035-003, Jackson) or peroxidase-conjugated goat anti-mice IgG (115–035-003, Jackson) depending on the primary antibody). Protein was quantified with Lab image Software (BioRad, USA) and expressed as relative units to housekeeping proteins.

The primary antibodies are as follows: anti-free fatty acid receptor 2 (FFAR2; ABC299, Merck Millipore), anti-monocarboxylic acid transporter 1 (MCT1, ab93048, Abcam), anti-small peptide transporters (PEPT1, ab203043, Abcam), anti-GAPDH (A01020, Abbkine), and anti-β-tubulin (A01030HRP, Abbkine).

### Measurement of mRNA by real-time quantitative PCR (RT-qPCR)

The total RNA were extracted from the hypothalamus and white adipose tissue using TRIzol agent, and then, reversed transcription was used to generate cDNA according to supplier specifications (Code No. RR820Q/A/B, TAKARA, Dalian, China). RT-qPCR analysis was carried out as follows: the cDNA samples (2 µL) were used as a template for the subsequent PCR reaction using gene-specific primers (Supplementary Table S4). RT-qPCR was performed using Piko Real Software 2.2 (Piko Real 96, Thermo Scientific, America). Intestinal tight junction protein was measured, including Occludin and Claudin-2. Appetite-related neuropeptide are as follows: neuropeptide Y(NPY), agouti-related protein (AgRP), pro- opiomelanocortin (POMC), cocaine and amphetamine-regulated transcript (CART), and hypothalamic brain-derived neurotrophic factor (BDNF) and tyrosine hydroxylase (TH). Inflammation-related molecules are as follows: chemokine (C-X-C motif), ligand 1(CXCL1), interleukin 6 (IL-6), and tumor necrosis factor-α(TNF-α).

### Measurement of SCFAs

Three SCFAs were measured in total: acetic acid, propionic acid, and butyric acid from cecal contents. SCFAs were measured via high performance gas chromatography (GC) (Agilent 7890A; Agilent Technologies, Germany) with a GC auto sampler, with a 30 m × 0.25 mm × 0.25 µm DB-WAX column [27].

### Statistical analysis

Statistical analysis was conducted using the SPSS 22.0 software package and GraphPad Prism 9. Differences in body mass and food intake were compared between treatment groups using a repeated-measure ANOVA. Measurements of other indexes were compared using one-way ANOVAs and Kruskal–Wallis test with *P* < 0.05 (*P < 0.05, **P < 0.01, ***P < 0.001). Results were presented as means ± SEM.

## Results

Supplementation of inulin to sick elephant for 7 days showed recovery in colic and had a good appetite. Most of the feces were in normal shape, and occasionally, individual fecal balls were in long strips. There was no overlong food fiber in the fecal balls (Fig. 1A). The daily defecation volume is about 35 kg, which belongs to the normal range.

### Inulin made the colic elephant recovered and changed gut microbiota

The profiling of microbiota composition by 16S rRNA gene sequencing showed an increased alpha diversity of the post-inulin group, which was significantly higher than the pre-inulin group (Shannon index, Fig. 1B). PCoA based on Bray–Curtis distance showed major alterations of the microbial community structure in the post-inulin group, which was closer to the control group (Fig. 1C). Here, Firmicutes and Bacteroidetes were the most abundant phylum in all elephants, while the ratio of Firmicutes and Bacteroidetes was higher in the pre-inulin group. The proportion of Spirochaetota was increased after inulin administration (Fig. 1D). *Lachnospiraceae_UCG_009*,* Treponema*, and *Lachnospiraceae_XPB1014_group* were lower in the pre-inulin group than in the control group, while the *Selenomonas* was higher in the pre-inulin group (Fig. 1E). The heat map showed that the gut microbiota structure in the pre-inulin group was significantly different from those of the control or post-inulin groups, *Lachnospiraceae_AC2044_group* and *Fibrobacter*, and *Bacteroidales_UCG-001* were lower in the pre- inulin group (Fig. 1F). Linear discriminant analysis effect size (LEfSe) displayed that the content of *Lachnospiraceae_UCG_009* and *Treponema* significantly increased after inulin treatment (Fig. 1G). After inulin treatment, the concentration of acetic acid, propionic acid, and butyric acid in the feces of the sick elephant increased slightly (Supplementary Table S5).

### Transplantation of “Inulin-microbiota” improved immunity of mice

To verify the efficacy of “inulin-microbiota,” the fecal microbiota of the sick elephant before and after treatment were transplanted into mice (Fig. 2A). The results showed that, relative to the “standard” mice, the mice with “elephant-microbiota” decreased weight gain (Figs. 2B, D) and white adiposity tissue weight (amount of epididymal, mesenteric, and subcutaneous fat) (Fig. 2E). There was no significant difference in blood glucose among the three groups after fasting for 5 h (Fig. 2F). The colon mass (Fig. 2G) and villus length (Fig. 2H, I) were lower in pre-FMT mice, largely reflecting the decline of the host’s ability to digest cellulose. “Inulin-microbiota” increased enterocyte proliferation, as measured by the number of PCNA-positive cells per villus in the proximal colon section (Fig. 2H, J).

**Fig. 3:**
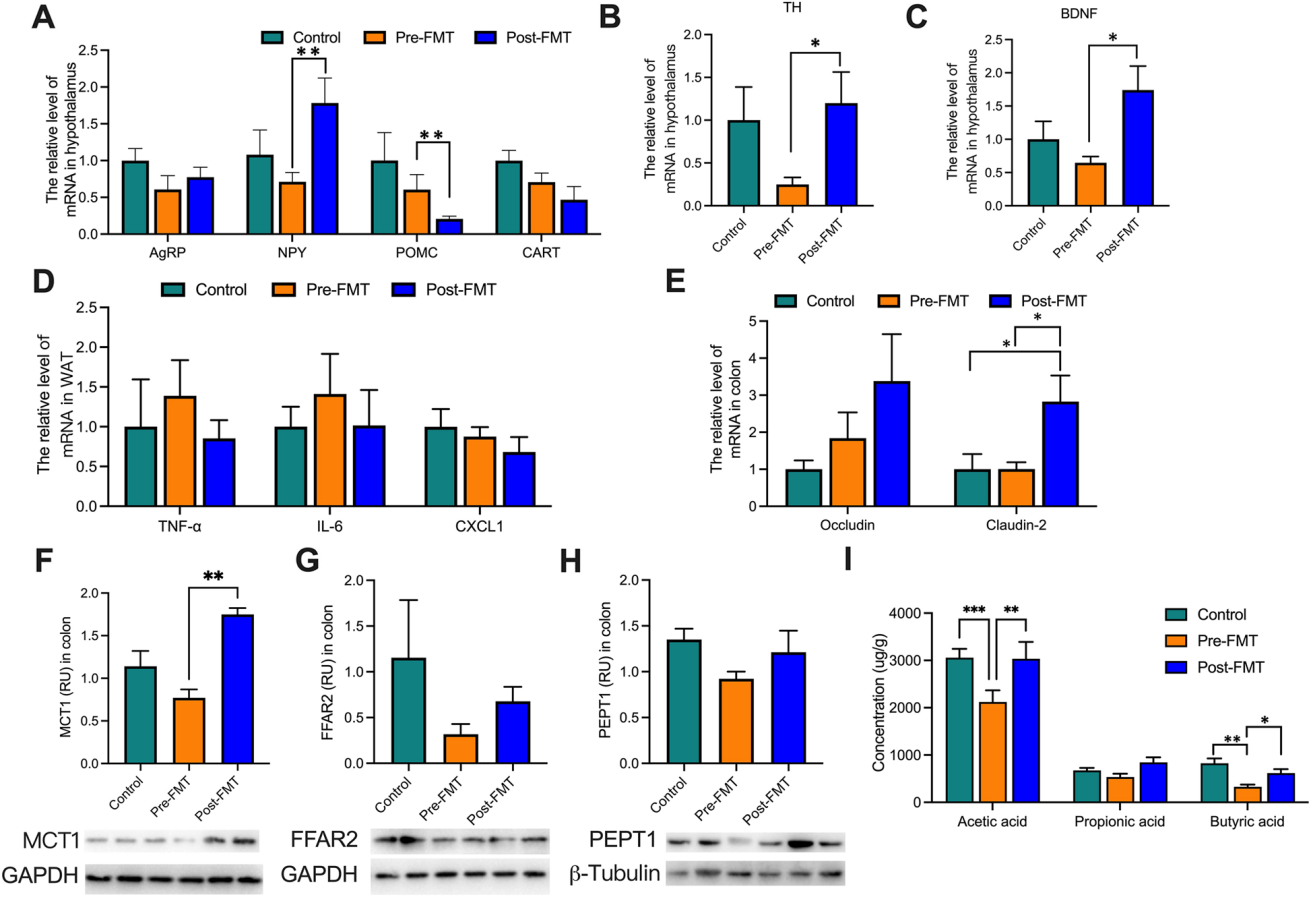
“Inulin-microbiota” affects immunity and nutrient transports in mice by FMT. A The mRNA was extracted from hypothalamus to analyze the expression of NPY, POMC, AgRP, and CART. B, C Quantitation of TH and BDNF in hypothalamus. D Quantitation of CXCL1, TNF-a, and IL-6 in white adipose tissue (WAT). E The relative expression of Occludin and Claudin-2 in the colon was analyzed by RT-PCR. F, G The relative expression of MCT1 and FFAR2 in the colon was analyzed by Western blotting. H Concentration of three short-chain fatty acids. Control: mice gavaged with mice fecal microbiota; Pre-FMT: mice gavaged with fecal microbiota from elephant before inulin treatment; Post-FMT: mice gavaged with fecal microbiota from elephant after inulin treatment. Data are means ± SEM. *P < 0.05, **P < 0.01, and ***P < 0.001

Although there was no significant difference in food intake among the three groups (Fig. 2C), NPY in the post-FMT mice was significantly higher than that in the pre-FMT mice, and POMC in the post-FMT mice was significantly lower than that in the Pre-FMT mice (Fig. 3A). At the same time, the contents of TH and BDNF in the hypothalamus increased significantly in the post-FMT group (Fig. 3B, C).

Furthermore, “inulin microbiota” reduced some indicators related to inflammation, such as CXCL1, TNF- a, and IL-6 (Fig. 3D) and increased the expression of colonic tight junction protein: Occludin and Claudin-2 (Fig. 3E). To explore the intestinal absorption of nutrients, the short-chain fatty acid receptor FFAR2 and transporter MCT1 were measured. The results showed that the content of colonic MCT1 in post-FMT mice increased significantly (Fig. 3F–H). The concentration of acetic acid and butyric acid in the pre-FMT group was significantly lower than the post-FMT group (Fig. 3I). Thus, these results suggest that inulin can improve the concentration of SCFAs and intestinal immune barrier, thereby reducing microbiota invasion and proinflammatory gene expression, which may be the basis of the protective effect of inulin on colic.

### Transplantation of “inulin-microbiota” changes the microbiota and physiological function of mice

**Fig. 4:**
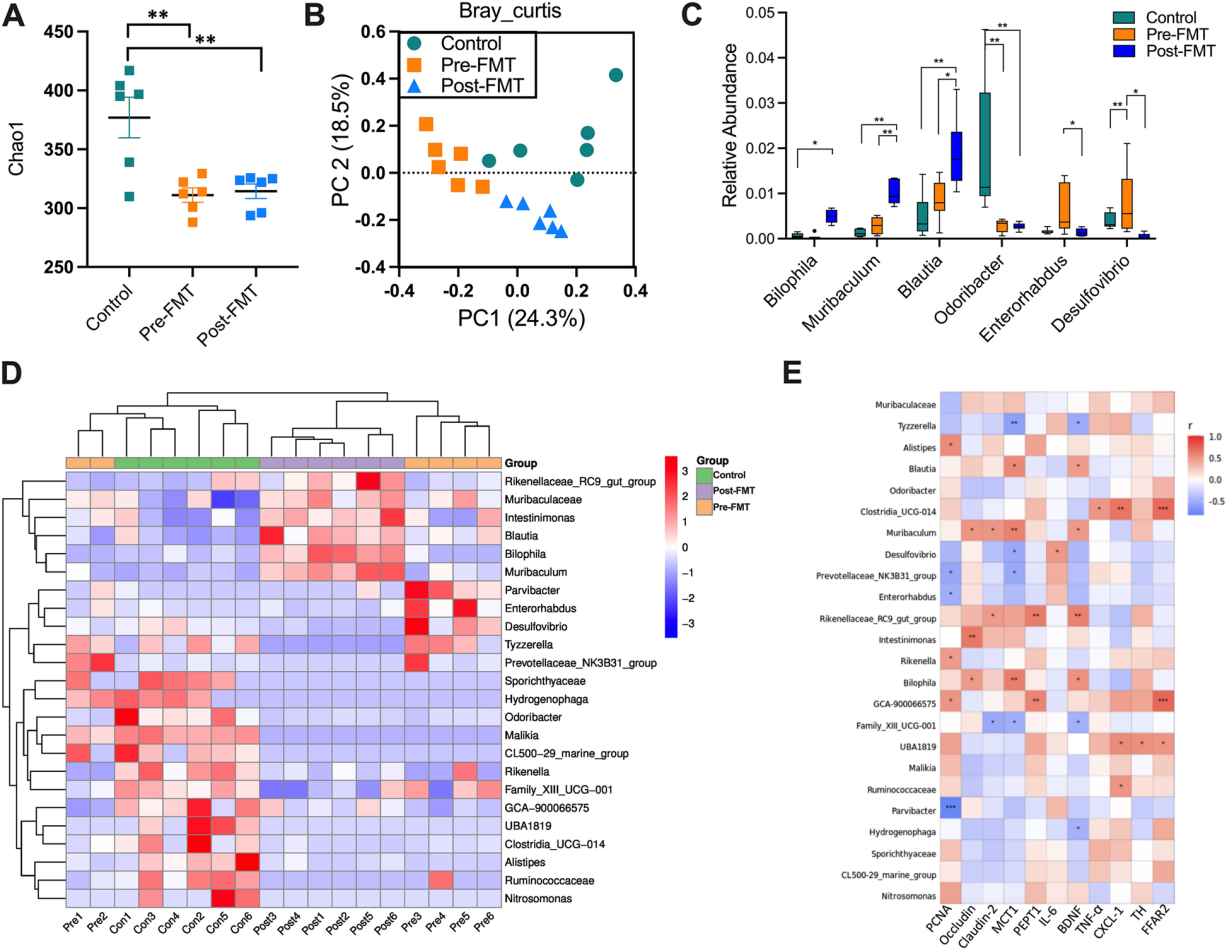
Changes of gut microbiota in mice after FMT. **A** Alpha diversity (Chao1) of bacterial communities across groups. **B** PCoA plot based on Bray–Curtis distance metrics in different groups. **C** Relative abundance of specific genus significantly altered by FMT. **D** Cluster heatmap showing the proportions of ASVs classified at the genus rank. E Correlation analysis between genus abundance and physiological indexes (Spearman). Control: mice gavaged with mice fecal microbiota; Pre-FMT: mice gavaged with fecal microbiota from elephant before inulin treatment; Post-FMT: mice gavaged with fecal microbiota from elephant after inulin treatment. Data are means ± SEM. *P < 0.05, **P < 0.01, and ***P < 0.001

The results showed that the gut microbiota after transplantation was similar to that of the host elephant (Supplementary Fig. 1A, B). Compared with the control group, the alpha diversity of mice transplanted with elephant feces generally decreased (Fig. 4A, Supplementary Fig. 1C). PCoA based on Bray–Curtis distance showed that there were significant differences among the three groups (ANOSIM, *P* = 0.001, Fig. 4B, Supplementary Fig. 1D). The difference of characteristic bacteria among the three groups at the genus level was also obvious (Fig. 4C). *Muribaculaceae*,* Blautia*,* Muribaculum*,* Oscillibacter*,* Bilophila*, and *Rikenellaceae_RC9* were enriched in the post-FMT group. *Enterorabdus* and *Desulfovibrio* were enriched in the pre-FMT group (Figs. 4C, D, Supplementary Fig. 1E). Correlation analysis between genus abundance and physiological indexes showed Blautia, Muribaculum, and Bilophila were positively correlated with colonic tight junction protein: Occludin and Claudin-2 also positively correlated with the SCFA transporter MCT1. Desulfovibrio was negatively correlated with the MCT1 (Fig. 4E).

## Discussion

### Inulin changed gut microbiota and relieved colic in Asian elephants

In recent years, with the development of metagenomics, the research related to the gut microbiota of Asian elephants has attracted much attention. The gut microbiota of free-ranging Asian elephants has been found to that Firmicutes account for the largest proportion, followed by Bacteroides, Proteobacteria, Fibrobacterota, Spirochaetota, and Actinobacteriota [28], while the gut microbiota of captive Asian elephants was mainly Firmicutes, Bacteroidetes, Proteobacteria, and Spirochaetota [29]. In this study, the gut microbiota of a sick Asian elephant was analyzed, and it was found that microbial alpha diversity and Fibrobacterota were decreased, and Firmicutes was much higher than other healthy captive elephants. Several beneficial commensal bacteria (e.g., *Lachnospiraceae_UCG_009* and *Lachnospiraceae_XPB1014_group*) were decreased. However, several proinflammatory bacteria (e.g., *Streptococcus*) were increased of the sick elephant. In the current study, the reduction of cellulose decomposing bacteria weakened the elephant’s ability to decompose dietary fiber, resulting in colitis. After inulin treatment, the gut microbiota composition of the sick elephant was gradually similar to that of other healthy elephants, with beneficial bacteria increasing and pathological symptoms disappearing.

Previous studies have demonstrated that prebiotics, such as oligosaccharides and inulin, can alleviate the body weight and fat accumulation by changing the gut microbiota [20]. Specifically, inulin increased the Firmicutes/Bacteroidetes ratio in individuals on a high-fat diet, which was thought to contribute to lose in weight, and reduced Proteobacteria and increased *Bifidobacteria*, which were reduced the chronic inflammatory [30]. Adding inulin to dairy cows’ diets can increase the abundance of beneficial symbiotic bacteria and improve the levels of amino acids and energy metabolism [31]. In the study of mice, the inulin diet increased *Bifidobacterium *spp. and *Anaerostipes caccae* (the beneficial microbes) and reduced harmful microbes like *Clostridium *spp. and *Escherichia-Shigella *spp*.* [32]. In the current study, inulin increased the richness of Bacteroidota and decreased Firmicutes and Actinobacteriota. Many bacteria in Bacteroides ferment dietary fiber to produce propionate, including *Prevotella*, *Bacteroidales BS11 gut group*, *Bacteroidales RF16 group*, and *Muribaculaceae* [33, 34]. A higher abundance of *Lachnospiraceae_UCG_009* and *Treponema* was observed in the after inulin treatment of sick elephant. These two bacteria and their metabolites promoted the formation of the intestinal mucus layer, helped to maintain the integrity of the intestinal barrier, and keep intestine health [35]. Therefore, inulin improved digestion and fecal status by adjusting the composition and structure of gut microbiota in elephant with colic.

### Inulin maintained intestinal barrier and reduced inflammation

According to previous studies, the transplantation of giant panda’s feces into mice provides a good model for study the physiological function of the intestinal microbiota of rare wild animals [36]. Thus, to verify the effect of “inulin-microbiota” on the physiology and digestion of elephants, elephant feces were transplanted into mice. Due to differences in diet and physiological characteristics of digestive tract, the intestinal microbiota of elephants seems to be more suitable for high-fiber feed, which was not very consistent with the diet of mice. Therefore, the weight and fasting blood glucose of mice transplanted with elephant microbiota were decreased. The current results showed that the villis of the colon were increased, the proliferation of colon cells were increased, and the nutrient absorption were improved in “inulin-microbiota” mice. The body fat of “inulin-microbiota” mice decreased, which is consistent with the therapeutic effect of inulin on mice fed with a high-fat diet [30]. Additionally, “inulin microbiota” was found to slightly reduced some indicators related to inflammation, such as CXCL1, TNF-a, and IL-6, and increased the tight junction proteins (Occludin and Claudin-2), which indicates the enhanced protection of the mucosal barrier. Occludin and Claudin-2 can reduce colonic permeability, thus reducing serum LPS and inflammatory cytokines (IL-1 β And IL-6) to reduce inflammation [37]. Similar studies showed that supplementation of inulin in cows’ diet could decrease the concentration of IL-6, IL-8, and TNF-α and increased acetic acid and butyric acid production [31]. SCFAs have an anti-inflammatory effect [38] and promote the recovery of the gut environment [39-41] and act directly or indirectly on the brain through hormonal, immune, and neural pathways [42]. Studies in mice also showed that SCFA could increase B10 cells and enhance the function of immunity [43]. To evaluate the transport and absorption capacity of SCFAs of “inulin microbiota” mice, the SCFAs receptor FFAR2, and transporter MCT1 were measured. We found that the content of MCT1 in the colon of mice transplanted with “inulin microbiota” increased significantly, indicating that the utilization rate of SCFA was increasing. In addition, the enriched bacteria in the “inulin microbiota” transplanted mice, such as *Blautia*,* Muribaculum*, and *Bilophila*, were also positively correlated with MCT1 and colon tight junction proteins. Therefore, we speculated that inulin improved the utilization of SCFA to strengthen the mucosal barrier and reduce inflammation and improved the digestive syndrome.

## Conclusions

This study reported the composition and structure of gut microbiota of elephant with colic. For the first time, through cross-species fecal transplantation, the current study presents new insight into the mechanism of inulin treatment of colic in elephants. The mechanism was found in mice as follows: inulin—gut microbiota—SCFAs—immune barrier. For animals, the use of prebiotics avoids the side effects of drugs and drug resistance caused by the abuse of antibiotics. Therefore, prebiotics would play an important role in disease prevention and treatment of captive animals in the future. However, this study still has significant limitations. We cannot determine whether the function of the gut microbiota transplanted into mice can fully represent the microbiota in elephants. The number of animal samples with diseases in this study is small and highly targeted. In the future, accumulating more cases can help provide data for other captive animals. Meanwhile, further research is needed on the effects of prebiotics on wild animals, like the types, dosage of prebiotics, and individual differences.

## Supplementary Information

**Additional file 1.** 

**Additional file 2.** 

**Additional file 3.** 

**Additional file 4.** 

## Data Availability

The raw sequencing data generated in this study have been deposited in the NCBI Sequence Read Archive under the accession numbers PRJNA818065 and PRJNA849734.

## Abbreviations

SCFAs: Short-chain fatty acids
FMT: Fecal microbiota transplant
PCNA: Proliferating cell nuclear antigen
FFAR2: Free fatty acid receptor 2
MCT1: Monocarboxylic acid transporter 1
PEPT1: Small peptide transporter
NPY: Neuropeptide Y
AgRP: Agouti-related protein
POMC: Pro-opiomelanocortin
CART: Cocaine and amphetamine-regulated transcript
BDNF: Brain-derived neurotrophic factor
TH: Tyrosine hydroxylase
CXCL1: Chemokine (C-X-C motif) Ligand 1
IL-6: Interleukin 6
TNF-α: Tumor necrosis factor-α
