# Supplementary material for: Mechanism of inulin in colic and gut microbiota of captive Asian elephant
Source: Microbiome. 2023 Jul 6;11:148. doi: 10.1186/s40168-023-01581-3 (PMC10324157; doi:10.1186/s40168-023-01581-3)
Supplement: Supplementary file 2 — Additional file 1. [file 40168_2023_1581_MOESM1_ESM.pdf]

Supplementary tables

Table S1. Daily ration of food categories of Asian elephants

Food categories	Mass (kg)
Pellets	9.75
Apple	18.75
Carrot	20
Banana	6.5
Cucumber	0.625
Fresh grass	200
Hay	37.5

Table S2. Nutrient analysis of diet composition on a dry matter basis

Macro-nutrients	Value
Crude protein	13.91%
Crude fat	3.16%
Crude fibre	27.12%
Carbohydrate	49.46%
Ash	7.03%
Gross energy	606538.25KJ

Table S3. Fecal samples of Asian elephants.

Name	Gender	Diet	Sample date
YINAN	Female	Mixed feed + Inulin	15,16,17/Oct;<br>22,23,24/Oct (after Inulin)
ALALIYA	Female	Mixed feed	16/Oct; 23,24/Oct
ZANLAN	Female	Mixed feed	16/Oct; 23,24/Oct
WANGNANJIAO	Female	Mixed feed	16/Oct; 23,24/Oct
MIGAILA	Female	Mixed feed	16/Oct; 23,24/Oct

Table S4. Primer sequence for qPCR.

NAME	PRIMERS	OLIGONUCLEOTIDES
Occludin	Forward primer	ATGTCCGGCCGATGCTCTC
Occludin	Reverse primer	TTTGGCTGCTCTTGGGTCTGTAT
Claudin-2	Forward primer	GTCATCGCCCATCAGAAGAT
Claudin-2	Reverse primer	ACTGTTGGACAGGGAACCAG
NPY	Forward primer	CGCTCTGCGACACTACATCAA
NPY	Reverse primer	GGGCTGGATCTCTTGCCAT
AgRP	Forward primer	GGTGCTAGATCCACAGAACCG
AgRP	Reverse primer	CCAAGCAGGACTCGTGCAG
POMC	Forward primer	CGAGGCCTTTCCCCTAGAGT
POMC	Reverse primer	CCAGGACTTGCTCCAAGCC

CART	Forward primer	TGGATGATGCGTCCCATGA
CART	Reverse primer	CGGAATGCGTTTACTCTTGAGC
BDNF	Forward primer	GCCTCCTCTACTCTTTCTGC
BDNF	Reverse primer	ATGGGATTACACTTGGTCTC
TH	Forward primer	CTACTGTCTGCCCGTGATT
TH	Reverse primer	CGGCTGGTAGGTTTGATC
CXCL1	Forward primer	TTGTGCGAAAAGAAGTGCAG
CXCL1	Reverse primer	TACAAACACAGCCTCCCACA
IL-6	Forward primer	GTGGCTAAGGACCAAGACCA
IL-6	Reverse primer	GGTTTGCCGAGTAGACCTCA
TNF-α	Forward primer	CGAGTGACAAGCCTGTAGCC
TNF-α	Reverse primer	CATGCCGTTGGCCAGGA
36B4（internal standard）	Forward primer	TCCAGGCTTTGGGCATCA
36B4	Reverse primer	CTTTATTCAGCTGCACATCACTCAGA

Table S5. Concentration of three short-chain fatty acids of Asian elephants.

	Pre-inulin	Post-inulin	Con	F	P value
Acetic acid (μ g/g)	1862±275.3	2484±163.6	2265±182.1	1.22	0.3295
Propionic acid (μ g/g)	922.1±169.4	1203±126	1008±62.88	1.407	0.2825
Butyric acid (μ g/g)	240.8±55.66	329.6±30.81	319.1±31.87	0.9846	0.4018
