# Supplementary figures and images for: Mechanism of inulin in colic and gut microbiota of captive Asian elephant
Source: Microbiome. 2023 Jul 6;11:148. doi: 10.1186/s40168-023-01581-3 (PMC10324157; doi:10.1186/s40168-023-01581-3)

A

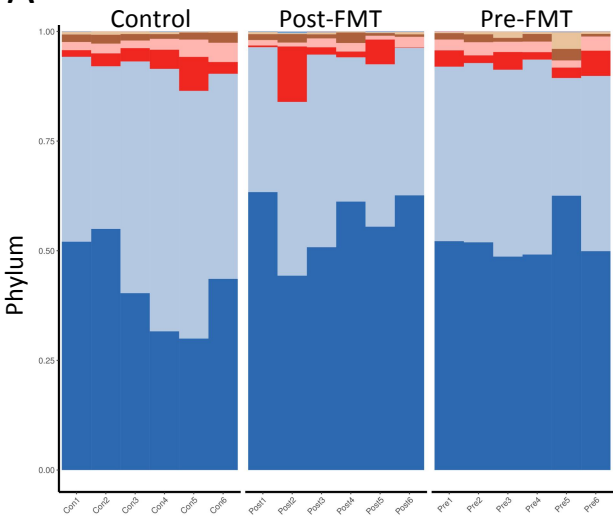

B

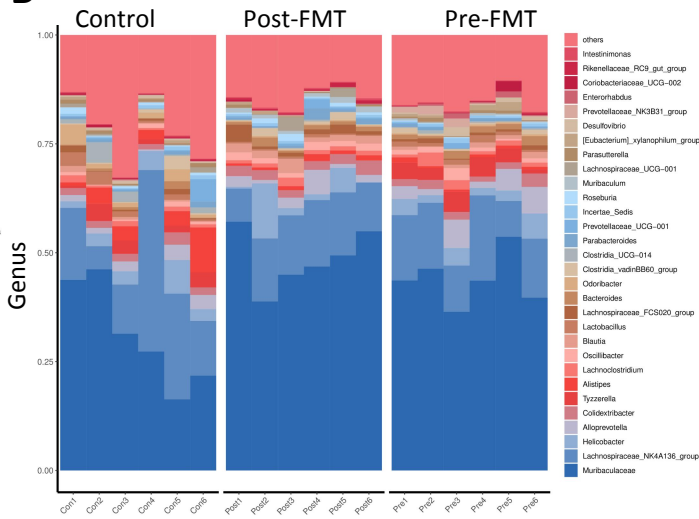

C

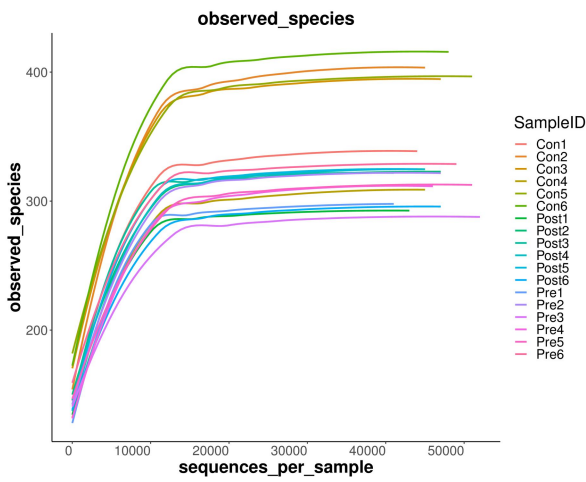

D

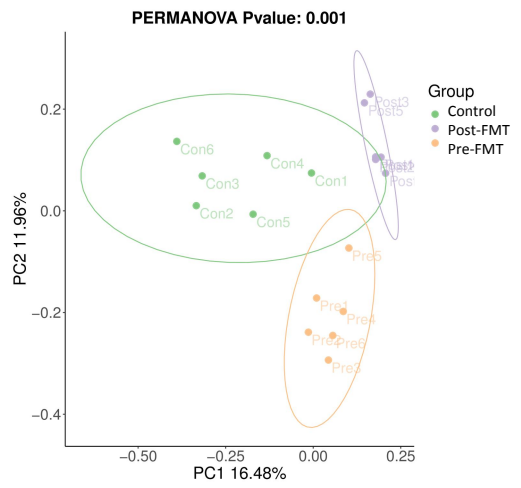

E

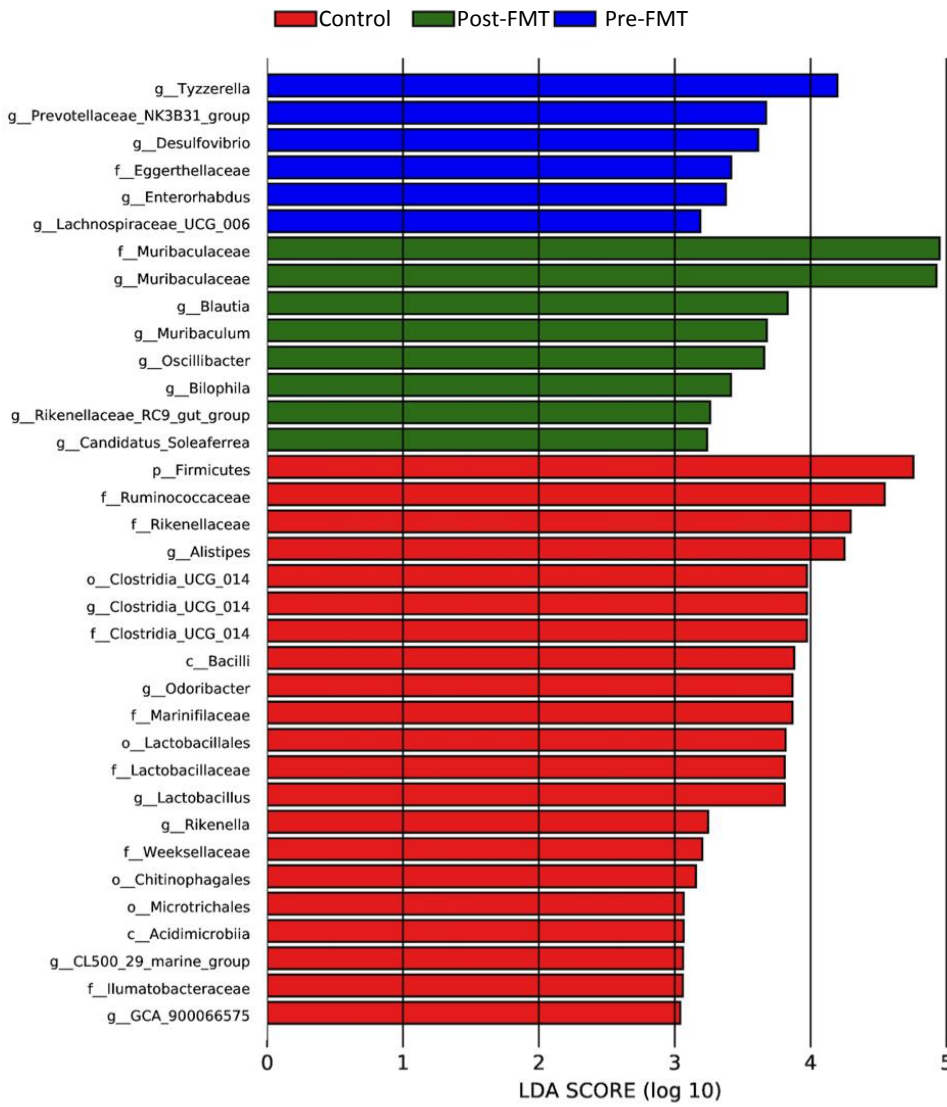

Supplement: Supplementary file 3 — Additional file 2. [file 40168_2023_1581_MOESM2_ESM.pdf]
