# Supplementary material for: Mechanism of inulin in colic and gut microbiota of captive Asian elephant
Source: Microbiome. 2023 Jul 6;11:148. doi: 10.1186/s40168-023-01581-3 (PMC10324157; doi:10.1186/s40168-023-01581-3)
Supplement: Supplementary file 4 — Additional file 3. [file 40168_2023_1581_MOESM3_ESM.pdf]

Urine-fecal test report form of Beijing Zoo Veterinary Hospital

Animal name： Asian elephant	Animal number： YINAN	Animal sex: Female	Animal age: Adult
Inspection type： faeces	Specimen status：dark brown, faint smell, multi fiber	Date received: 2021.10.7 am	Completion date: 2021.10.7 am
Veterinarian for examination: Yi LUO	Inspector: Minghai YANG	Verifierz: Minghai YANG	Final adjudicator: Tianchun PU

	Urine test items	Results		Stool examination test items	Results
1	Glu	/	1	OB	-
2	Bil	/	2	Mucus	-
3	Ket	/	3	PC	-
4	SG	/	4	Ova	-
5	PH	/	5	CPV\CCV	/
6	Pro	/	6	FCoV	/
7	URO	/	7	FPV	/
8	NIT	/	42	CDV/CAV	/
9	BLO	/	44	Intestinal microbiota activity	
10	WBC	/	45		
11	ALB	/	46		
12	CRE	/	47		
13	A:C	/	48		

Result analysis

No abnormality is found in the test

Note: This result is only responsible for this sample. Clinical staff should make diagnosis according to the actual situation.

Biochemical routine test report form of Beijing Zoo Veterinary Hospital

Animal species：Asian elephant	Animal number：YINAN	Animal sex: Female	Animal age: Adult
Inspection type：blood	Specimen status：normal	Date received: 2021.10.14	Completion date: 2021.10.15
Veterinarian for examination: Yi LUO	Inspector: Xiangxiang LI	Verifierz: Yunsheng WANG	Final adjudicator: Tianchun PU

Inspection items	Results	Tips	Units	Reference data
HGB	152		g/L	100~155
RBC	3.86		*10^12/L	1.98~4.00
WBC	13.8		*10^9/L	6.40~15.00
HCT	0.41		L/L	0.30~0.44
NS	0.46			0.20~0.50
NST	0.07			
EO	0.01	↓		0.06~0.15
BA	0			0~0.02
LY	0.41			0.40~0.75
MO	0.05			0.02~0.08
K	4.1		mmol/L	4.6±0.6
Na	135		mmol/L	130±5
Cl	91		mmol/L	89±4
Ca	2.49		mmol/L	2.65±0.23
P	1.73		mmol/L	1.62±0.39
GLU	3.54	↓	mmol/L	5.106±1.166
CR	158.29		μmol/L	141±35
BUN	3.20	↓	mmol/L	4.641±1.428
UA	3.82		μmol/L	12±18
ALT	2.20		U/L	8±9
TP	76.36		g/L	81±8
Alb	32.70		g/L	33±5
GLB	43.66		g/L	49±9
A/G	0.7			±
AST	10.24	↓	U/L	22±11
TBA	3.44		μmol/L	
CHE	131.59		U/L	
TBIL	3.54		μmol/L	3±3
DBIL	2.81		μmol/L	2±2
