# Supplementary material for: Mechanism of inulin in colic and gut microbiota of captive Asian elephant
Source: Microbiome. 2023 Jul 6;11:148. doi: 10.1186/s40168-023-01581-3 (PMC10324157; doi:10.1186/s40168-023-01581-3)
Supplement: Supplementary file 5 — Additional file 4. [file 40168_2023_1581_MOESM4_ESM.pdf]

Pathological log

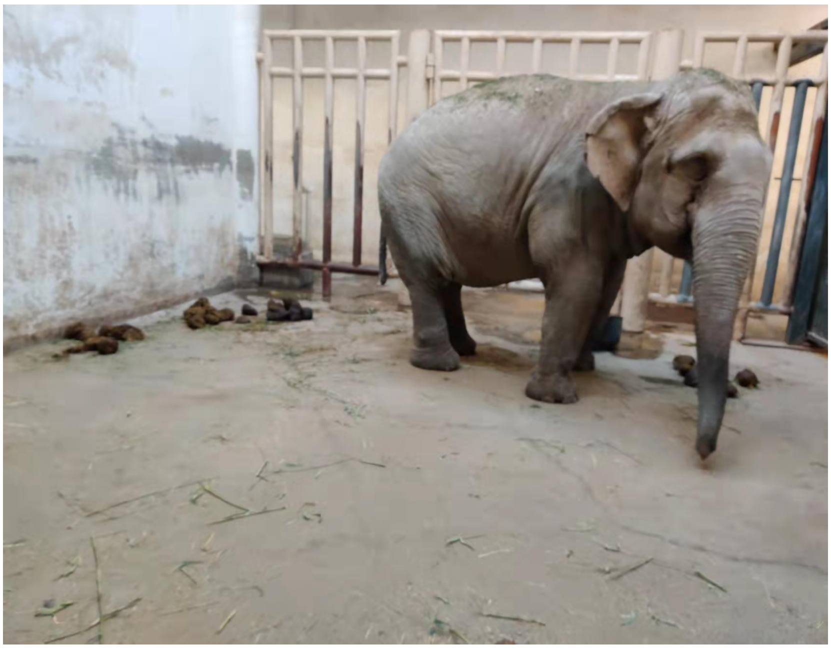

October 13, 2021

Today, there were 4 big beach fecal balls, which were ball shaped, and the weight of feces reached 40kg, and the feed intake was increasing. There was a large amount of undigested plant fibers in the feces.

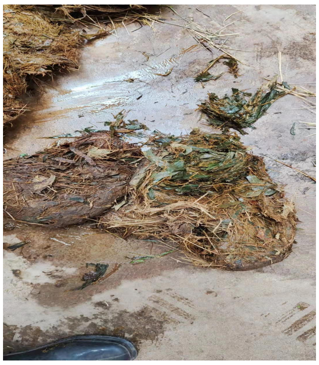

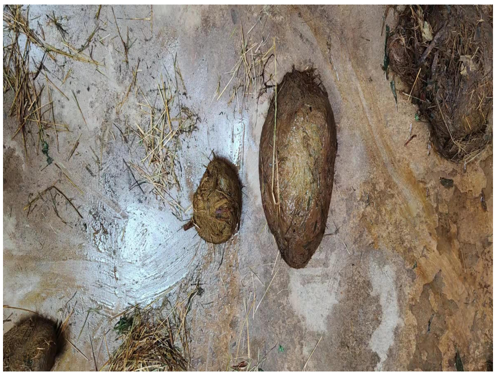

October 14, 2021

The amount of feces reached 36kg. Feces were round, with undigested plant fibers inside, with a length of 10 cm. It indicates that the gastrointestinal tract and the digestive function is abnormal.

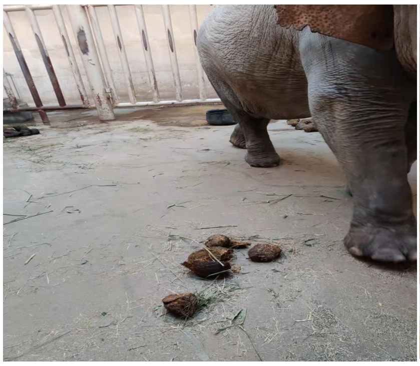
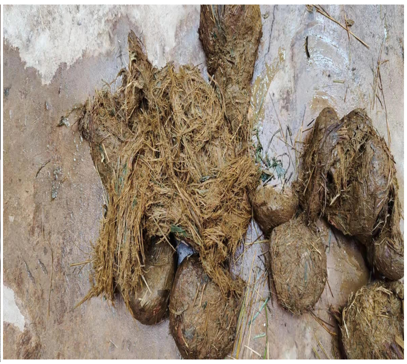
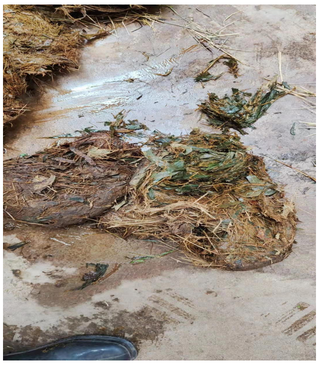
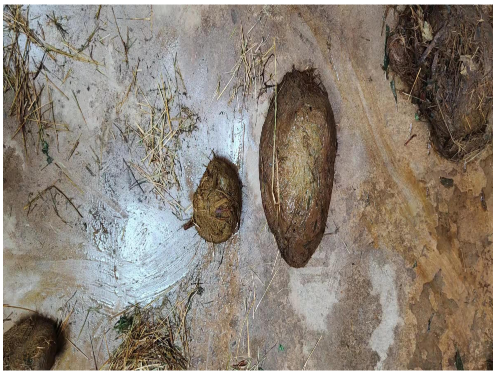

October 20, 2021

Start taking inulin, 500g per elephant per day.

October 23, 2021

500g inulin per day.

The elephant had a good appetite. The shape of feces was mostly

normal, and indigestible plant fibers still exist in feces. The daily defecation volume is about 35kg in the normal range.

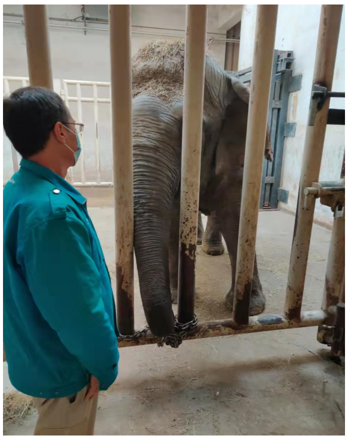 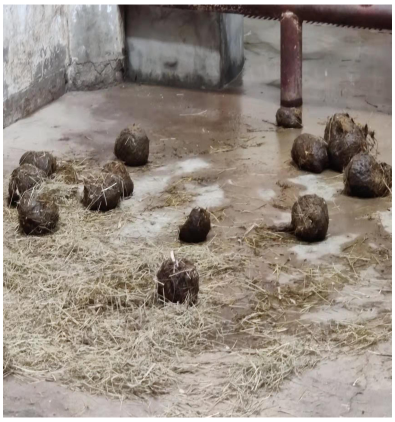

October 25, 2021

The elephant was in good condition, and the feces were formed. Occasionally, the feces were in long strips, and there were no long fibers in the feces. Good appetite.

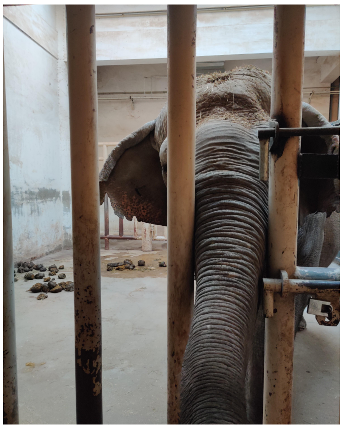 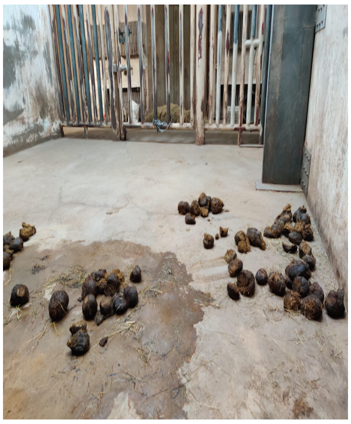 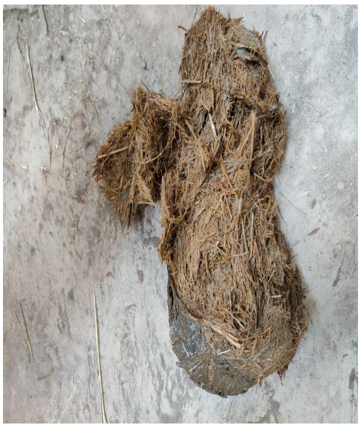

November 12, 2021

Recently, the elephant has performed well, with normal spirit and appetite.Most of the feces are spherical, and only a few grow in strips. Stop feeding inulin.
